# Psychiatric health care need in Hungary identified by the short screening algorithm of depression and suicide risk used in general medical practices

**DOI:** 10.1038/s41598-023-41437-2

**Published:** 2023-08-31

**Authors:** Judit Diószegi, Zoltán Rihmer, Péter Torzsa, László Pál, Árpád Czifra, Xenia Gonda, János Sándor

**Affiliations:** 1 Department of Public Health and Epidemiology, Faculty of Medicine, University of Debrecen, Kassai út 26, Debrecen, 4028 Hungary; 2 Department of Psychiatry and Psychotherapy, Faculty of Medicine, Semmelweis University, Balassa utca 6, Budapest, 1082 Hungary; 3 National Institute of Mental Health, Neurology and Neurosurgery, Amerikai út 57, Budapest, 1145 Hungary; 4 Department of Family Medicine, Faculty of Medicine, Semmelweis University, Stáhly u. 7-9, Budapest, 1085 Hungary; 5 ELKH-DE Public Health Research Group, Department of Public Health and Epidemiology, Faculty of Medicine, University of Debrecen, Kassai út 26, Debrecen, 4028 Hungary

**Keywords:** Diseases, Health care, Risk factors

## Abstract

Suicides are often related to depression. General medical practices (GMPs) should play a role in screening depression. We aimed to test the screening algorithm of Rihmer and Torzsa for depression and suicide and determine the prevalence and number of patients in the nationwide representative Hungarostudy 2002 population, and to estimate the corresponding extra health care need in an average GMP and in the Hungarian population in addition to patients who are already cared for by specialized care. The short version of the Beck Hopelessness Scale (BHS) and the Hungarian version of the short form of the Beck Depression Inventory (BDI-9) were used to screen for suicide risk and depression. The prevalence of suicidal thoughts and depression was determined and findings were extrapolated to an average GMP of 1,600 adults and to the population over 25 years of age. This screening would generate a considerable extra psychiatric care to organize and implement in an average GMP and throughout the country. Our findings show that with easily administered screening instruments a significant number of patients likely to have depression can be identified at the primary care level, arguing for the establishment of the extra psychiatric care capacity in Hungary.

## Introduction

Although being preventable in many cases with appropriate psychiatric treatment, suicide accounted for more than 700,000 deaths worldwide in 2019 with males and older people as well as persons with major mental disorders being overrepresented among suicide decedents, coupled by an approximately twenty times higher frequency of suicide attempts. Despite the substantial decrease in deaths from suicides of over 40% during the past two decades in the European Region, the highest crude death rates (12.8 per 100,000 population) were still reported in this region among all the regions of the World Health Organization (WHO)^1^. Though a remarkably decline was observed in Hungary as well, Hungary still ranked as the 21st country in the world and the 10th in the European Region by suicide mortality^2^.

Suicide is a complex phenomenon influenced by an interplay of several medical-psychiatric, psycho-social, demographic and cultural factors. Still the majority of suicides are associated with psychiatric disorders. Accordingly, 60–98% of all deaths from suicides are reported to be related to mental disorders with depression being the most common followed by substance-use related disorders, schizophrenia, and personality disorders^3–8^.

According to estimates of the Health Interview Survey (EHIS 2019), 8% of the Hungarian population aged 15 and over is affected by depressive symptoms during a one year period, 4% of them declared having this health condition, and 5% suffered from depressive symptoms according to the Personal Health Questionnaire Depression Scale (PHQ-8) during the 2 weeks prior to questioning^9^. Using the most strict diagnostic criteria of officially declared mental disorders in a representative sample of the adult Hungarian population, the lifetime, 1-year and point prevalence of DSM-III-R major depressive disorder was 15.1%, 7.1% and 2.6%, respectively^10^. According to two independent Hungarian studies, the point prevalence of major depressive disorder in primary care practices were 5% and 7.3%, respectively^11, 12^. The importance of depression is underlined by the fact that ten to fifteen per cent of untreated severe depression patients die by suicide (about 50% of them attempts it) and 65–75% of suicides deaths are related to major depression being mostly untreated or undertreated^4, 13^.

Less than half of the patients with depression in a general medical practice (GMP) seek medical care, and the majority of those, who do, seek medical advice from their general practitioners (GPs)^13^. Considering the high prevalence of depression in primary care and the strong relationship between unrecognized/untreated depression and suicide^14^, as well as the common medical contact made by suicide victims a few weeks before their suicidal act, GPs play an important role in the recognition and management of suicide risk^4, 12, 13, 15^. Furthermore, since suicidal behavior often develops in a later course, (in more severe stage) of depression, early detection and effective treatment of currently non-suicidal depressive patients, frequently seen in primary care, can be considered as a hidden but undoubtedly effective form of suicide prevention^4, 16^.

Several guidelines recommend, as part of a stepped care approach, screening, identification and even treatment of depression at the level of primary care^17^, and recently this need has been articulated in Hungary as well, which would have several advantages. Beyond decreasing both the stigmatization associated with depression and psychiatric treatment, as well as decreasing the burden on secondary, specialist psychiatric care, this approach is likely to improve the efficacy of identifying patients with depression and increase the ratio of those getting adequate treatment, with divergent benefits, including increased well-being and quality of life as well as functionality of these patients, decreased societal and economic costs resulting from untreated depression^18, 19^, and also likely decrease the prevalence of suicide attempts and deaths from suicides.

**Figure 1:**
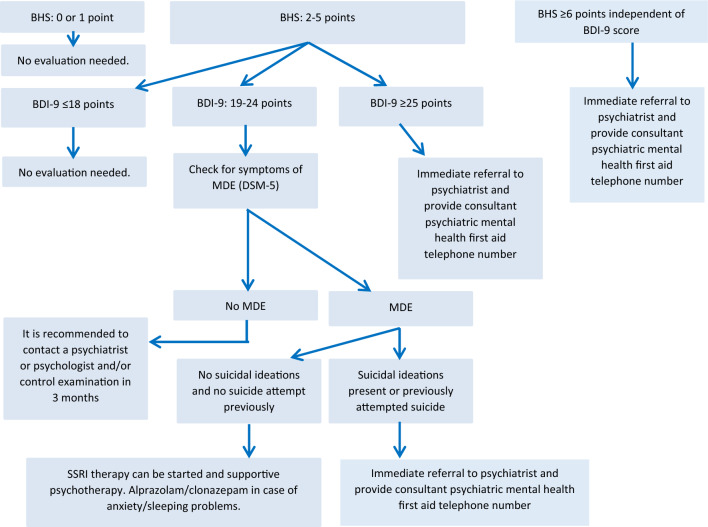
Screening and treatment algorithm of depression and suicide in general medical practices^15^. *BHS* Beck Hopeless Scale short version (four-item). *BDI*-9 Beck Depression Inventory short version (nine-item), *MDE* major depressive episode.

Using the short version of the Beck Hopelessness Scale (BHS, four-item, Table 1) and the short form of the Beck Depression Inventory consisting of nine items (BDI-9, Table 2), acute suicide risk and the presence of a major depressive episode can be most likely determined in GMPs as well. Figure 1 summarizes the screening process and treatment options of depression and suicide in GMPs described by Rihmer and Torzsa^15^.

**Table 1:** Items of the short version of the Beck Hopelessness Scale (four-item).

1. My future seems dark to me
2. Things just won’t work out the way I want them to
3. There’s no use in really trying to get something I want because I probably won’t get it
4. I feel that the future is hopeless and that things cannot improve

**Table 2:** Items of the short version of the Beck Depression Inventory (nine-item).

1. I have lost all of my interest in other people
2. I can’t make decisions at all anymore
3. I wake up several hours earlier than I used to and cannot get back to sleep
4. I am so worried about my physical problems that I cannot think of anything else
5. I am too tired to do anything
6. I am so worried about my physical problems that I cannot think of anything else
7. I feel the future is hopeless and that things cannot improve
8. I am dissatisfied or bored with everything
9. I feel guilty all of the time

Early detection and treatment of depression and suicide risk is of high priority. Therefore, the aim of our study was to investigate the original screening and treatment algorithm for depression and suicide developed by Rihmer and Torzsa^15^ as well as a modified version with different cut-off values in a large, representative general adult population sample, to determine the umber and prevalence of patients, who need health care and to estimate the corresponding extra health care need generated by the screening algorithm in an average GMP and in the Hungarian population over 25 years of age.

## Methods

### Study design and sampling

This investigation is a secondary data processing national study based on the Hungarostudy 2002 survey, a cross-sectional survey representing the adult Hungarian population according to sex, age, and sub-regions. This national survey aimed to investigate the physical and mental wellbeing of the Hungarian population, psychosocial risk factors and the problems and needs related to health care services. Details of the cluster randomized sampling are described elsewhere^20, 21^. The refusal rate was 17.7%. Altogether 12,668 individuals (0.16% of the Hungarian adult population) completed the four-item version of the Beck Hopelessness Scale (BHS) and the Beck Depression Inventory consisting of 9 items. Participants were interviewed at their homes (5666 males and 6987 females, sex data was missing for 15 individuals; mean age: 47.59 ± 17.87) by healthcare professionals (members of the Hungarian Health Visitor Network).

Our study was a secondary analysis. It utilized the opportunity that the Hungarostudy determined Beck Depression Inventory and Beck Hopelessness Scale, and combined application of these scales for suicide risk assessment could be tested. This large representative database was the most recent available dataset containing both type of psychometric scales.

There was no primary data collection in this study. The present paper has been produced using the anonymised database without personal identifiers provided for our research group by the Hungarostudy Research Group. According to the Hungarian legislation (Act CLV of 2016 on Official Statistics entered into force on 1 January 2017^22^ and by Government Decree 184/2017^23^ issued for the implementation of this law), there was no need for ethical approval to implement the secondary analysis of the database. Primary data collection was implemented in the Hungarostudy 2002, which study was approved by the Ethical Committee of the Semmelweis University, Budapest, Hungary (Ref. No. 13/2002) and conforms to the principles embodied in the Declaration of Helsinki.

### Short version of the Beck Hopelessness Scale

Hopelessness is an independent and strong predictor of suicide risk^24, 25^. The short version of the beck hopelessness scale (four-item, Table 1) was applied to investigate hopelessness, with a cut-off value of 6 for suicide risk. The BHS can be characterized by 100% sensitivity and 71% specificity^26^ and the short (four-item) BHS has been found to have excellent psychometric properties compared with the original 20-item scale. The internal consistency is excellent (Cronbach’s alpha: 0.85)^27^. Answers were evaluated on a four-point Likert scale ranging from 0 to 3 (0–not typical; 1–rarely typical; 2–typical; 3–very typical)^27^. Total scores of 6–8 indicate suicide risk and total scores of 9 or greater indicate that very high suicide risk warrants consultation with a psychiatrist or referral to psychiatric admission is needed^15^.

### Hungarian version of the short form of the Beck Depression Inventory

The short form of the Beck Depression Inventory consisting of nine items was used to screen for depression (Table 2). Answers were evaluated on an intensity scale ranging from 1 to 4 (1–not typical; 2–rarely typical; 3–typical; 4–very typical). BDI-9 contains the following items: social withdrawal, indecisiveness, sleep disturbances, fatigability, somatic preoccupation, work difficulty, pessimism, dissatisfaction and self-accusation^28^. The following severity categories can be identified based on total scores of the inventory: scores between 9 and 13 indicate no depression; scores between 14 and 18 indicate likelihood of mild depression; scores from 19 through 24 indicate likelihood of moderate depression; and scores of 25 and above indicate likelihood of severe depression^15, 28^. BDI as a screening tool for depression in primary health care patients with a cut-off score of 19 was found to have a 68.6% sensitivity and 97.5% specificity by Rózsa et al.^28^.

### Screening algorithm

Figure 1 describes the screening algorithm and cut-off scores for screening and treatment options of depression and suicide in GMPs^15^. According to this algorithm, first the BHS should be completed and in case of a total score of 0 or 1 no further evaluation is needed. On the other hand, scores of 6 or greater indicate acute suicide risk and need for referral of the patients to a psychiatrist even without completing the BDI-9^27^. When patients score 2 to 5 on the Beck Hopelessness Scale, the Beck Depression Inventory should be completed. If the BDI-9 total scores are below 19, no further evaluation is needed. When BDI-9 total scores are between 19 and 24, patients should be evaluated for major depressive episode according to DSM-5 diagnostic criteria^29^. If either of the first two symptoms and additional four symptoms are present according to DSM-5, major depressive episode (MDE) can be diagnosed. Meanwhile, for patients with scores of 2–5 on the BHS and 19–24 on BDI-9, but not meeting major depression criteria, referral to a psychiatrist or psychologist should be considered (or close monitoring and repeated evaluation using the screening instruments is needed after 3 months). When patients meet DSM-5 criteria for major depressive episode and have BDI-9 total scores of 19–24, but no suicide risk is present (no suicidal ideations or plans and no history of previous suicide attempt), then treatment of non-suicidal depression can be started in GMPs as well^13, 30^. This treatment process should be supported by a clinical psychologist and should involve consultations or referral to a psychiatrist if needed. Treatment by a psychiatrist is necessary when patients score 19–24 on BDI-9, meet DSM-5 criteria for major depressive episode and suicidal ideations are present or the patient previously attempted suicide. Scores equal to 25 or higher on the Beck Depression Inventory indicate likelihood of major depression^12^. It is also advisable to provide the telephone number of mental health emergency services (S.O.S or hot-line crisis) when starting psychiatric care^15^.

### Modified screening algorithms

For the second algorithm the following modifications were introduced: BHS cut-off for “no further evaluation required” was changed from 0 or 1 point to 0–2 points. In line with these changes, BDI-9 completion is needed for patients with BHS scores of 3–5 (Supplementary Fig. 1). Furthermore, a third screening algorithm (Supplementary Fig. 4) was created with the need for BDI-9 assessment for patients scoring 4–5 points on BHS.

### Statistical analysis

The prevalence and the number patients with suicidal thoughts and major depression needing health care was determined in the Hungarostudy 2002 population following the categories of three screening algorithms. The observed proportions of the Hungarostudy 2002 were used to estimate the number of patients in an average GMP of 1600 adults and in the Hungarian population over 25 years of age (7,288,433 in 2018^31^), who need extra health care.

### Ethical approval

There was no primary data collection in this study. The present paper has been produced using the anonymised database without personal identifiers provided for our research group by the Hungarostudy Research Group. According to the Hungarian legislation (Act CLV of 2016 on Official Statistics entered into force on 1 January 2017 and by Government Decree 184/2017 issued for the implementation of this law), there was no need for ethical approval to implement the secondary analysis of the database. Primary data collection was implemented in the Hungarostudy 2002, which study was approved by the Ethical Committee of the Semmelweis University, Budapest, Hungary (Ref. No. 13/2002) and conforms to the principles embodied in the Declaration of Helsinki.

### Consent to participate

Not applicable. Because all data in our secondary analyses were unidentified and information potentially connectable to individuals was not utilized, informed consent was not required according to Hungarian legislation.

## Results

### Hungarostudy 2002 population

**Figure 2:**
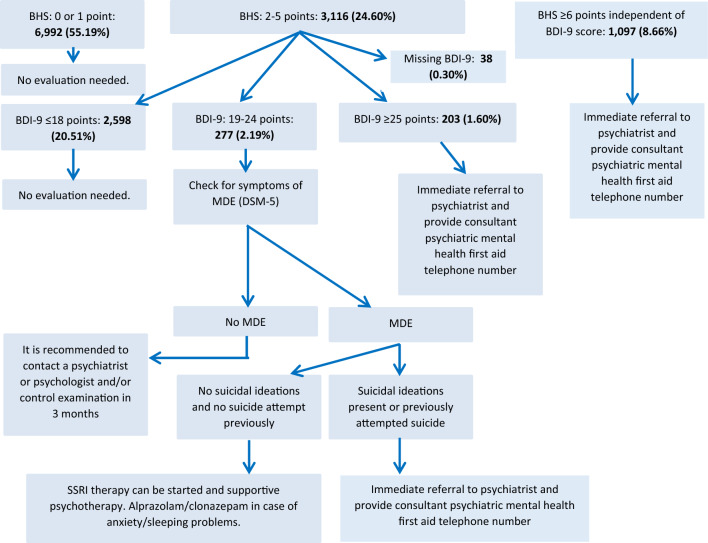
Prevalence and number of patients needing health care according to the screening algorithm of Rihmer & Torzsa^15^ in the Hungarostudy 2002 population. *BHS* Beck Hopeless Scale short version (four-item), *BDI*-*9* Beck Depression Inventory short version (nine-item), *MDE* major depressive episode.

The four-item version of the BHS and the BDI consisting of 9 items was completed by 12,668 individuals. Subjects with previously identified psychiatric conditions (n = 805, 6.35%) were excluded from the analysis. This was necessary because those patients were already evaluated by psychiatrists and diagnosed with psychiatric conditions; therefore, in their case screening cannot be implemented. BHS was not available for 658 individuals (5.19%). Among those, who completed BHS, BDI-9 could not be completed in case of 491 subjects. 1097 patients (8.66%) needed immediate referral to a psychiatrist and 6992 subjects (55.19%) needed no further evaluation. 3116 patients (24.60%) had had to be further evaluated according to BDI-9 scores. Figure 2 shows the number of cases in categories of the screening protocol^15^ in the Hungarostudy 2002 sample.

### Estimated yield of screening in an average general medical practice

**Figure 3:**
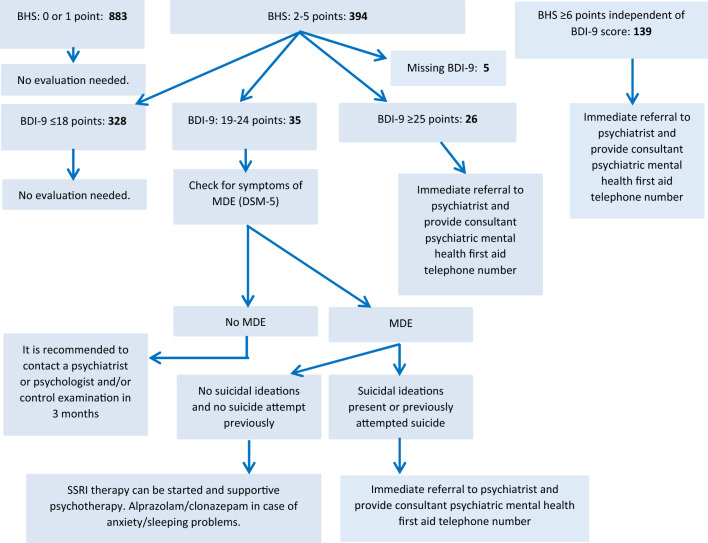
Estimated number of patients needing health care according to the screening algorithm of Rihmer & Torzsa^15^ in an average general medical practice (1600 patients). *BHS* Beck Hopeless Scale short version (four-item), *BDI*-9 Beck Depression Inventory short version (nine-item), *MDE* major depressive episode.

The number of patients in an average general medical practice who need extra health care was estimated based on the Hungarostudy 2002 population. Figure 3 shows the estimated number of cases in categories of the screening protocol^15^ in an average GMP with 1600 patients. The number of patients with previously identified psychiatric conditions and with missing BHS was estimated to be 102 and 83, respectively. It was calculated that 139 patients would meet the criterion for immediate referral to a psychiatrist due to acute suicide risk and 883 subjects would not need further evaluation. According to the screening protocol, BDI-9 evaluation was indicated for 394 individuals. Based on our results, psychiatric care was indicated for 165 patients in a 1600-patient GMP and there may be a need for care in case of additional group of 35 patients, while 1211 individuals would be healthy regarding depression and/or acute suicide risk.

### Estimated yield of screening in the Hungarian adult population

**Figure 4:**
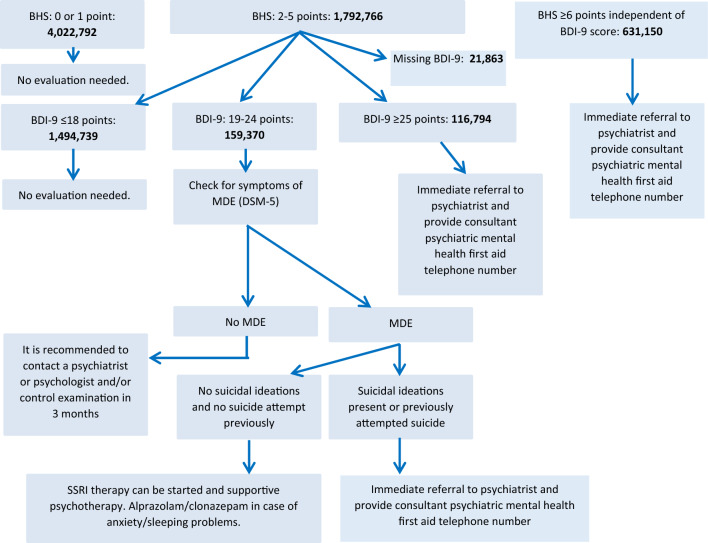
Estimated number of patients needing health care according to the screening algorithm of Rihmer & Torzsa^15^ in the Hungarian adult population (25 + years), 2018. *BHS* Beck Hopeless Scale short version (four-item), *BDI*-*9* Beck Depression Inventory short version (nine-item), *MDE* major depressive episode.

The number of patients in the Hungarian adult population needing extra health care was estimated based on the Hungarostudy 2002 population. Figure 4. shows the estimated number of cases in categories of the screening protocol^15^ in the Hungarian adult population over 25 years of age. The number of patients with previously diagnosed psychiatric conditions and with missing BHS scores was estimated to be 463,150 and 378,575 individuals, respectively. The number of individuals with BHS scores indicating suicide risk was calculated to be 631,150, and the number of subjects with BHS < 2 points and scores of 2–5 points was found to be 4,022,792 and 1,792,766, respectively. Based on these estimations psychiatric care is indicated for 747,944 individuals in the Hungarian adult population older than 25 years of age and there may be a potential need for care in case of additional 159,370 subjects, while 5,517,531 individuals would not be affected by depression and/or suicide risk.

### Modified protocols

Increasing the cut-off value of the BHS for the need of BDI-9 assessment, the prevalence and case numbers for categories of the protocol has changed the following way:

In the Hungarostudy 2002 population the number of individuals, who did not need further evaluation based on BHS total scores was 8169 (64.49%). Acute suicide risk (BHS ≥ 6) was identified for 1097 (8.66%) respondents. BDI-9 assessment was indicated for 1939 patients (15.39%) due to BHS scores of 3–5. Among them BDI-9 scores of 19–24 indicated the assessment of MDE according to DSM-5 criteria in case of 235 (1.86%) subjects. The necessity of immediate referral to a psychiatrist was identified for 178 patients (1.41%). Extrapolating these findings to an average GMP with 1600 patients and to the adult population of Hungary (2018) 25 years of age, 161 and 733,561 patients would need extra psychiatric health care definitively (Table 3); and additional 30 and 135,205 individuals potentially, respectively.

**Table 3:** Number of patients needing definitive psychiatric care based on Beck Hopelessness Scale (BHS) cut off-values.

BHS	Average sized general medical practice*	Hungarian adult population**
2–5 points	165	747,944
3–5 points	161	733,561
4–5 points	156	709,972

*1600 patients per general medical practice, **Hungarian 25 + years old population in 2018: 7,288,433.

According to the third protocol, in the Hungarostudy 2002 population, no further evaluation was needed in case of 8996 (71.01%) subjects, while 1097 (8.66%) individuals had BHS ≥ 6 scores. BHS scores of 4–5 indicated the need for further assessment for 1112 (8.78%) respondents. Among these subjects 190 (1.50%) had BDI-9 scores of 19–24 (assessment of MDE needed) and 137 (1.08%) were identified with BDI-9 ≥ 25 points (immediate referral to psychiatrist). Extrapolating these findings, 156 patients would need definitive psychiatric care in an average 1600-patient GMP and 709,972 individuals in the adult population of Hungary (2018) over 25 years of age (Table 3). Furthermore, additional 24 and 109,315 individuals may need psychiatric care, respectively.

Supplementary Figs. 1–6 present the number of cases in categories of the two modified screening protocols.

## Discussion

Our study tested mental health screening algorithms utilizable in primary care practices and investigated the prevalence and the number of patients suffering from depression and individuals with acute suicide risk, who would need health care based on the nationwide Hungarostudy 2002 survey. For the assessment of acute suicide risk and depression the four-item version of the Beck Hopelessness Scale and the short form of the Beck Depression Inventory consisting of nine items were used. Properties of the short version of the BHS were evaluated previously and found to be a quick and useful screening tool of suicidal risks in GMPs and in psychiatric specialist care^27, 32^. The nine-item BDI used for the evaluation of depression in our study was applied in several Hungarian epidemiological studies previously; therefore, medical specialists are familiar with it^15, 33, 34^. Accordingly, these screening tools can be integrated into the everyday work of general medical practices. This is the first time when these screening instruments were applied in a representative general population sample in Hungary. Furthermore, according to our estimations the proposed screening activity would generate a remarkable increase in workload for primary and secondary care, in addition to the patients who are already cared for, namely there is considerable extra psychiatric care to organize, coordinate and implement in an average Hungarian GMP (165–200 patients) and corresponding psychiatric catchment area, and throughout the country (747,944–907,314 patients). Even when following the modified protocols with increased cut-off values of BHS for the need of BDI-9 assessment, these numbers (161–191 or 156–180 patients in a GMP and 733,561–868,766 or 709,972–819,287 patients in the country) did not change markedly.

Based on our current knowledge, suicide and suicide attempts are predictable and preventable in many cases and several studies provided evidence that early detection and appropriate treatment of depression can be considered as essential and effective methods of suicide^4, 13, 35^. General practitioners play a particularly important role in this process, since it was shown that the introduction of postgraduate training programs for GPs on the diagnosis and treatment of depression significantly reduced the frequency of suicide, need for inpatient depression treatment and sick leave due to suicide attempts^13, 36–38^. Patients identified by the screening method would need further evaluation and treatment and should be referred to specialists when indicated. This applies to primary care settings as well, for which convincing evidence was found that screening for depression improves the accurate identification of adult patients suffering from this mental disorder and subsequent therapy decreases clinical morbidity^39^. Thus, involving primary practices in screening and treatment for depression is increasingly adopted by healthcare and mental healthcare guidelines. However, the implementation of screening for depression at the population level requires the reorientation of primary care services in Hungary^40^. Currently, the majority of primary health care services in Hungary are provided by single-handed GP practices consisting of a GP and a practice nurse^41–43^. Services of convectional single-handed GP practices are often limited to patient care and referral to a specialist^40^. Consequently, previous studies have found that single-handed GP practices are less efficient in providing preventive services including screening for mental disorders^41, 44^. Therefore, the creation of multidisciplinary group practices of self-employed physicians, practice nurses, health professionals, such as psychologists and health mediators have been suggested as a more effective alternative^40^. Result from a model program in Hungary demonstrated that multidisciplinary group practices were significantly more efficient in carrying out of health status assessment of registered clients than single-handed counterparts^41^. In addition, they were also more successful in screening for behavioral risk factors of disease among clients in disadvantaged populations^41^. To provide more equal and effective primary care services in Hungary, a government decree was approved in 2021 which requires single-handed GP practices to establish multidisciplinary group practices. This decree provides an opportunity to directly involve medical specialists in GMP’s work^45^. The system-wide reorientation of primary care will enable multidisciplinary group practices to provide lifestyle and behavioral counseling for patients and to identify those with a likelihood of depression and risk factors for suicide^41^. However, in order to carry out this task, primary care physicians should be equipped with easily and quickly administrable and reliable instruments to screen for depression and suicide risk as well as validated algorithms for guided decision. Based on our previous work and present results, the large-scale routine implementation of the four-item version of the BHS and the short form of the BDI for the assessment of acute suicide risk and depression in multidisciplinary group practices can be realistic within a few years being easily and quickly administrable and reliable. Though the availability of experts to evaluate screen-positive subjects is a major point is this process. The main goal would be to lower the suicide risk that could stay undetected (sensitivity); however, several non-threatened individuals could be included (specificity). We expect to detect individuals without serious suicide threat, and to screen all the individuals at risk.

It is unequivocal that screening is essential but in line with the recommendations it can only be implemented when adequate working capacity is ensured. Considering that the number of psychiatrists in Hungary was 14.9 per 100,000 inhabitants in 2018^46^ and that more than 150 Hungarian psychiatrists work abroad for time periods longer than 10–15 years, and many retired psychiatrists work only in part-time, in addition to the increased involvement of primary care providers and mental health professional such as psychologists in multidisciplinary care practices a corresponding increase in psychiatric care capacity is needed to be able to comply with the high number of cases who would need treatment for depression and increased suicide risk identified by the proposed screening protocol, in addition to patients already being treated.

Limitations must be considered when interpreting the findings of our study. There has been a long period of time between administration of the survey and the current project. Though this large representative database (Hungarostudy 2002 survey) was the most recent available dataset containing both type of psychometric scales applied. Demographic changes may have altered our results but it may be hypothesized that not to a large extent.

In conclusion our study evaluating screening algorithms proposed by Rihmer and Torzsa^15^ in a large representative general population sample revealed a remarkable extra health care need in an average general medical practice and at the population level, in relation to this, there is considerable extra psychiatric care to organize at the level of primary care and secondary care settings. As soon as the capacities can be built up for this task, the systematic application of the screening can begin in agreement with several healthcare guidelines recommending screening for depression in primary care, if there is available specialist care capacity to deal with the screened cases. Furthermore, the proposed screening algorithm allows the identification of patients at risk for suicide and even those who suffer from depressive episodes and are outside of specialized health care. Therefore, not only early treatment and referral to specialist is facilitated, which can ultimately reduce mortality and burden of depression at the country level, but this process also decreases the burden on secondary, specialist psychiatric care. Furthermore, since hopelessness is an independent and strong predictor of suicide risk, therefore, evaluating hopelessness with easily administered screening instruments, such as BHS, beyond screening for mental health disorders is an important component of suicide prevention. Factors and conditions associated with hopelessness deserve special attention, e.g. chronic physical illness, traumatic experiences and stressful life events (major childhood adverse events, for example, sexual abuse, discrimination, bullying)^47^. Thus the extended use of this screening instrument beyond the health care level may be considered and recommended.

### Supplementary Information

Supplementary Figures.

## Data Availability

Our investigation is a secondary analysis of anonymised data provided by the Hungarostudy 2002 Research Group after accepting their terms of use. The authors of this study agreed to use this database exclusively for non-profit research and cite the original work in any of their investigations/publications, where it is intended to be used. Therefore, ethical approval was not required for our study.
