# Supplementary material for: Psychiatric health care need in Hungary identified by the short screening algorithm of depression and suicide risk used in general medical practices
Source: Sci Rep. 2023 Aug 31;13:14249. doi: 10.1038/s41598-023-41437-2 (PMC10471575; doi:10.1038/s41598-023-41437-2)
Supplement: Supplementary file 1 — Supplementary Figures. [file 41598_2023_41437_MOESM1_ESM.docx]

**Psychiatric health care need in Hungary identified by the short screening algorithm of depression and suicide risk used in general medical practices**

Judit Diószegi, Zoltán Rihmer, Péter Torzsa, László Pál, Árpád Czifra, Xenia Gonda, János Sándor

**Supplementary Figure 1. Prevalence and number of patients needing health care according to the second screening algorithm of Rihmer & Torzsa (2016) in the Hungarostudy 2002 population**

BHS ≥6 points, independent of BDI-9 score: **1,097 (8.66%)**

Immediate referral to psychiatrist and provide consultant psychiatric mental health first aid telephone number

BHS: 0, 1 or 2 points:  **8,169 (64.49%)**

No evaluation needed.

No evaluation needed.

No MDE

BDI-9 ≤18 points: **1,495 (11.80%)**

No suicidal ideations and no suicide attempt previously

Suicidal ideations present or previously attempted suicide

Check for symptoms of MDE (DSM-5)

BDI-9: 19-24 points: **235 (1.86%)**

BDI-9 ≥25 points: **178 (1.41%)**

BHS: 3-5 points: **1,939 (15.31%)**

Immediate referral to psychiatrist and provide consultant psychiatric mental health first aid telephone number

SSRI therapy can be started and supportive psychotherapy. Alprazolam/clonazepam in case of anxiety/sleeping problems.

MDE

Immediate referral to psychiatrist and provide consultant psychiatric mental health first aid telephone number

It is recommended to contact a psychiatrist or psychologist and/or control examination in 3 months

Missing BDI-9: **31 (0.24%)**

BHS: Beck Hopeless Scale short version (four-item)

BDI-9: Beck Depression Inventory short version (nine-item)

MDE: major depressive episode

**Supplementary Figure 2. Estimated number of patients needing health care according to the second screening algorithm of Rihmer & Torzsa (2016) in an average general medical practice (1600 patients)**

BHS ≥6 points, independent of BDI-9 score: **139**

Immediate referral to psychiatrist and provide consultant psychiatric mental health first aid telephone number

BHS: 0, 1 or 2 points:  **1,032**

BHS: 3-5 points: **245**

No evaluation needed.

No evaluation needed.

No MDE

BDI-9 ≤18 points: **189**

No suicidal ideations and no suicide attempt previously

Suicidal ideations present or previously attempted suicide

Check for symptoms of MDE (DSM-5)

BDI-9: 19-24 points:  **30**

BDI-9 ≥25 points:  **22**

Immediate referral to psychiatrist and provide consultant psychiatric mental health first aid telephone number

SSRI therapy can be started and supportive psychotherapy. Alprazolam/clonazepam in case of anxiety/sleeping problems.

MDE

Immediate referral to psychiatrist and provide consultant psychiatric mental health first aid telephone number

It is recommended to contact a psychiatrist or psychologist and/or control examination in 3 months

Missing BDI-9: **4**

BHS: Beck Hopeless Scale short version (four-item)

BDI-9: Beck Depression Inventory short version (nine-item)

MDE: major depressive episode

**Supplementary Figure 3. Estimated number of patients needing health care according to the second screening algorithm of Rihmer & Torzsa (2016) in the Hungarian adult population (25+ years), 2018**

BHS ≥6 points, independent of BDI-9 score: **631,150**

Immediate referral to psychiatrist and provide consultant psychiatric mental health first aid telephone number

BHS: 0, 1 or 2 points: **4,699,969**

No evaluation needed.

No evaluation needed.

No MDE

BDI-9 ≤18 points: **860,136**

No suicidal ideations and no suicide attempt previously

Suicidal ideations present or previously attempted suicide

Check for symptoms of MDE (DSM-5)

BDI-9: 19-24 points: **135,205**

BDI-9 ≥25 points: **102,411**

BHS: 3-5 points: **1,115,588**

Immediate referral to psychiatrist and provide consultant psychiatric mental health first aid telephone number

SSRI therapy can be started and supportive psychotherapy. Alprazolam/clonazepam in case of anxiety/sleeping problems.

MDE

Immediate referral to psychiatrist and provide consultant psychiatric mental health first aid telephone number

It is recommended to contact a psychiatrist or psychologist and/or control examination in 3 months

Missing BDI-9: **17,836**

BHS: Beck Hopeless Scale short version (four-item)

BDI-9: Beck Depression Inventory short version (nine-item)

MDE: major depressive episode

**Supplementary Figure 4. Prevalence and number of patients needing health care according to the third screening algorithm of Rihmer & Torzsa (2016) in the Hungarostudy 2002 population**

BHS ≥6 points, independent of BDI-9 score: **1,097 (8.66%)**

Immediate referral to psychiatrist and provide consultant psychiatric mental health first aid telephone number

BHS: 0 - 3 points:  **8,996 (71.01%)**

No evaluation needed.

No evaluation needed.

No MDE

BDI-9 ≤18 points: **767 (6.05%)**

No suicidal ideations and no suicide attempt previously

Suicidal ideations present or previously attempted suicide

Check for symptoms of MDE (DSM-5)

BDI-9: 19-24 points: **190 (1.50%)**

BDI-9 ≥25 points: **137 (1.08%)**

BHS: 4-5 points: **1,112 (8.78%)**

Immediate referral to psychiatrist and provide consultant psychiatric mental health first aid telephone number

SSRI therapy can be started and supportive psychotherapy. Alprazolam/clonazepam in case of anxiety/sleeping problems.

MDE

Immediate referral to psychiatrist and provide consultant psychiatric mental health first aid telephone number

It is recommended to contact a psychiatrist or psychologist and/or control examination in 3 months

Missing BDI-9: **18 (0.14%)**

BHS: Beck Hopeless Scale short version (four-item)

BDI-9: Beck Depression Inventory short version (nine-item)

MDE: major depressive episode

**Supplementary Figure 5. Estimated number of patients needing health care according to the third screening algorithm of Rihmer & Torzsa (2016) in an average general medical practice (1600 patients)**

BHS ≥6 points, independent of BDI-9 score: **139**

Immediate referral to psychiatrist and provide consultant psychiatric mental health first aid telephone number

BHS: 0 - 3 points:  **1,136**

No evaluation needed.

No evaluation needed.

No MDE

BDI-9 ≤18 points: **97**

No suicidal ideations and no suicide attempt previously

Suicidal ideations present or previously attempted suicide

Check for symptoms of MDE (DSM-5)

BDI-9: 19-24 points:  **24**

BDI-9 ≥25 points:  **17**

BHS: 4-5 points: **140**

Immediate referral to psychiatrist and provide consultant psychiatric mental health first aid telephone number

SSRI therapy can be started and supportive psychotherapy. Alprazolam/clonazepam in case of anxiety/sleeping problems.

MDE

Immediate referral to psychiatrist and provide consultant psychiatric mental health first aid telephone number

It is recommended to contact a psychiatrist or psychologist and/or control examination in 3 months

Missing BDI-9: **2**

BHS: Beck Hopeless Scale short version (four-item)

BDI-9: Beck Depression Inventory short version (nine-item)

MDE: major depressive episode

**Supplementary Figure 6. Estimated number of patients needing health care according to the third screening algorithm of Rihmer & Torzsa (2016) in the Hungarian adult population (25+ years), 2018**

BHS ≥6 points, independent of BDI-9 score: **631,150**

Immediate referral to psychiatrist and provide consultant psychiatric mental health first aid telephone number

BHS: 0 -3 points: **5,175,777**

No evaluation needed.

No evaluation needed.

No MDE

BDI-9 ≤18 points: **441,287**

No suicidal ideations and no suicide attempt previously

Suicidal ideations present or previously attempted suicide

Check for symptoms of MDE (DSM-5)

BDI-9: 19-24 points: **109,315**

BDI-9 ≥25 points: **78,822**

BHS: 4-5 points: **639,780**

Immediate referral to psychiatrist and provide consultant psychiatric mental health first aid telephone number

SSRI therapy can be started and supportive psychotherapy. Alprazolam/clonazepam in case of anxiety/sleeping problems.

MDE

Immediate referral to psychiatrist and provide consultant psychiatric mental health first aid telephone number

It is recommended to contact a psychiatrist or psychologist and/or control examination in 3 months

Missing BDI-9: **10,357**

BHS: Beck Hopeless Scale short version (four-item)

BDI-9: Beck Depression Inventory short version (nine-item)

MDE: major depressive episode
